# 1-Aza­niumyl­cyclo­butane-1-carboxyl­ate monohydrate

**DOI:** 10.1107/S1600536813033217

**Published:** 2014-01-29

**Authors:** Ray J. Butcher, Greg Brewer, Aaron S. Burton, Jason P. Dworkin

**Affiliations:** a Department of Chemistry, Howard University, 525 College Street NW, Washington, DC 20059, USA; b Department of Chemistry, Catholic University of America, Washington, DC 20064, USA; c NASA Johnson Space Center, Astromaterial and Exploration Science Directorate, Houston, TX 77058, USA; d Solar System Exploration Division, NASA Goddard Space Flight Center, Greenbelt, MD 20771, USA

## Abstract

In the title compound, C_5_H_9_NO_2_·H_2_O, the amino acid is in the usual zwitterionic form involving the α-carboxyl­ate group. The cyclo­butane backbone of the amino acid is disordered over two conformations, with occupancies of 0.882 (7) and 0.118 (7). In the crystal, N—H⋯O and O—H⋯O hydrogen bonds link the zwitterions [with the water molecule involved as both acceptor (with the NH_3_^+^) and donor (through a single carboxylate O from two different aminocyclobutane carb­oxylate moities)], resulting in a two-dimensional layered structure lying parallel to (100).

## Related literature   

For the eighty amino acids that have been detected in meteorites or comets, see: Burton *et al.* (2012 ▶); Pizzarello *et al.* (2004 ▶), (2006 ▶). For the role of the H atom on the α-C atom in enhancing the rate of racemization, see: Yamada *et al.* (1983 ▶). For the mechanism of racemization of amino acids lacking an α-H atom, see: Pizzarello & Groy (2011 ▶). For the role that crystallization can play in the enrichment of l-isovaline and its structure, see: Butcher *et al.* (2013 ▶). For normal bond lengths and angles, see: Orpen (1993 ▶). For the hydro­chloride salt of the title compound and related non-proteinogenic amino acids, see: Chacko & Zand (1975 ▶); Butcher *et al.* (2013 ▶); Brewer *et al.* (2013 ▶). For conformational studies on model proteins with 1-amino­cyclo­butane-1-carb­oxy­lic acid residues, see: Balaji *et al.* (1995 ▶). For involvement of the title compound in ethyl­ene production that leads to the ripening and spoilage of fruit, see: Nakatsuka *et al.* (1998 ▶); Bulantseva *et al.* (2003 ▶). 
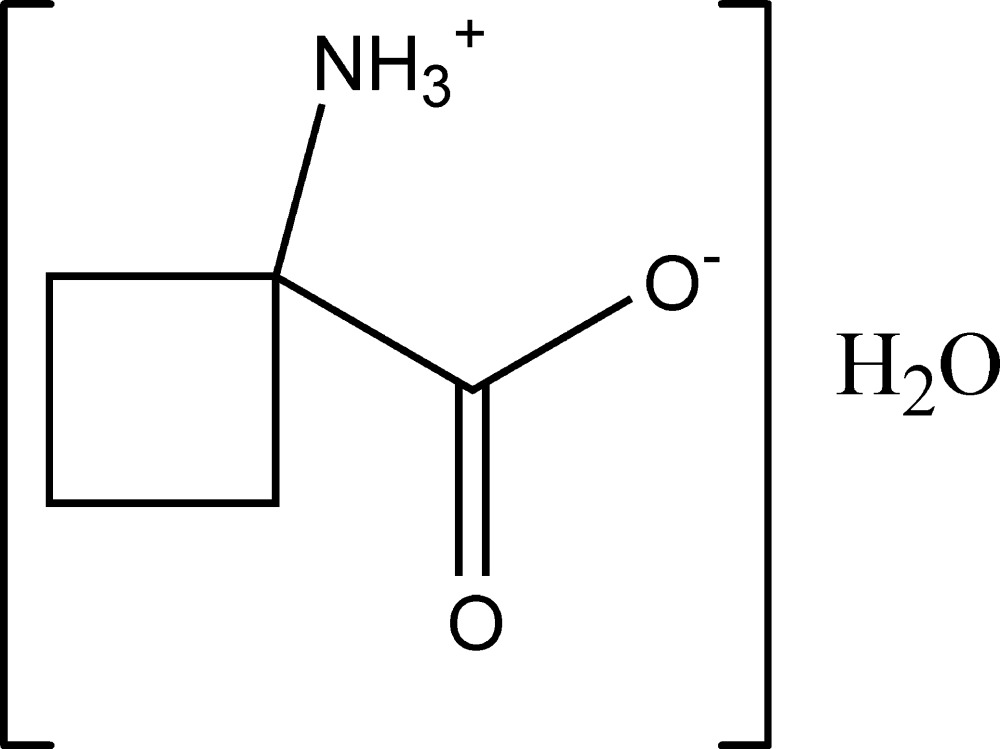


## Experimental   

### 

#### Crystal data  

C_5_H_9_NO_2_·H_2_O
*M* *_r_* = 133.15
Monoclinic, 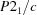

*a* = 10.25082 (19) Å
*b* = 6.13117 (9) Å
*c* = 10.9209 (2) Å
β = 100.8735 (18)°
*V* = 674.05 (2) Å^3^
*Z* = 4
Cu *K*α radiation
μ = 0.92 mm^−1^
*T* = 123 K
0.41 × 0.34 × 0.16 mm

#### Data collection  

Agilent Xcalibur (Ruby, Gemini) diffractometer
Absorption correction: multi-scan (*CrysAlis PRO*; Agilent, 2012 ▶) *T*_min_ = 0.784, *T*_max_ = 1.000
4405 measured reflections
1400 independent reflections
1360 reflections with *I* > 2σ(*I*)
*R*_int_ = 0.022

#### Refinement  

*R*[*F* ^2^ > 2σ(*F* ^2^)] = 0.038
*wR*(*F* ^2^) = 0.103
*S* = 1.06
1400 reflections
119 parameters
H atoms treated by a mixture of independent and constrained refinement
Δρ_max_ = 0.36 e Å^−3^
Δρ_min_ = −0.19 e Å^−3^

### 

Data collection: *CrysAlis PRO* (Agilent, 2012 ▶); cell refinement: *CrysAlis PRO*; data reduction: *CrysAlis PRO*; program(s) used to solve structure: *SHELXS97* (Sheldrick, 2008 ▶); program(s) used to refine structure: *SHELXL97* (Sheldrick, 2008 ▶); molecular graphics: *SHELXTL* (Sheldrick, 2008 ▶); software used to prepare material for publication: *SHELXTL*.

## Supplementary Material

Crystal structure: contains datablock(s) I, New_Global_Publ_Block. DOI: 10.1107/S1600536813033217/zs2282sup1.cif

Structure factors: contains datablock(s) I. DOI: 10.1107/S1600536813033217/zs2282Isup2.hkl

Supporting information file. DOI: 10.1107/S1600536813033217/zs2282Isup3.cml

CCDC reference:

Additional supporting information:  crystallographic information; 3D view; checkCIF report

## Figures and Tables

**Table 1:** Hydrogen-bond geometry (Å, °)

*D*—H⋯*A*	*D*—H	H⋯*A*	*D*⋯*A*	*D*—H⋯*A*
O1*W*—H1*W*1⋯O1^i^	0.87 (2)	1.92 (2)	2.7935 (12)	175.2 (18)
O1*W*—H1*W*2⋯O2^ii^	0.82 (2)	2.01 (2)	2.8268 (12)	175.7 (19)
N1—H1*N*⋯O2^iii^	0.922 (18)	1.923 (18)	2.8087 (12)	160.6 (15)
N1—H2*N*⋯O1^iv^	0.930 (17)	1.913 (17)	2.8351 (12)	171.2 (14)
N1—H3*N*⋯O1*W*	0.902 (17)	1.895 (17)	2.7673 (13)	162.3 (15)

Symmetry codes: (i) 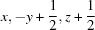; (ii) 
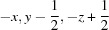
; (iii) 
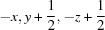
; (iv) 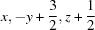.

**Fig. 1.**
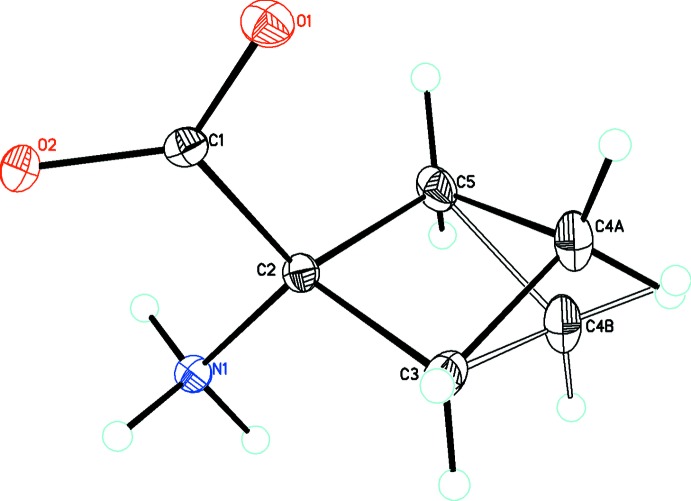
Fif. 1. Diagram of the title compound showing atom labeling. Atomic displacement parameters are at the 30% probability level. The disorder in the backbone is shown with atoms in the the minor component connected with unfilled bonds. Hydrogen bonds are shown as dashed lines.

**Fig. 2.**
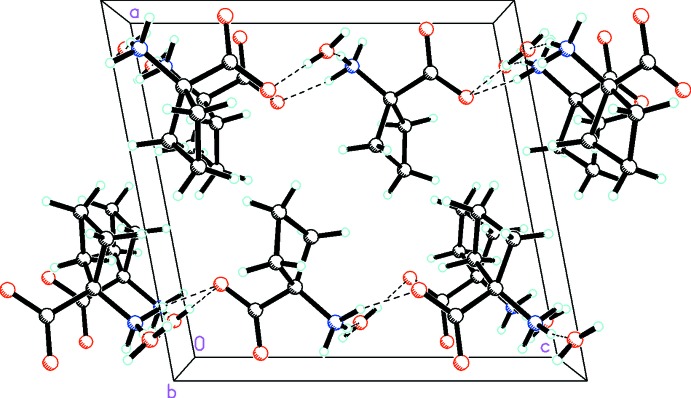
Packing diagram of the title compound (major component only) viewed along the b axis showing the N—H···O and O—H···O hydrogen bonds as dashed lines.

## supplementary crystallographic information

### 1. Comment

The alpha amino acids are essential for life as they are the building blocks of all proteins and enzymes. Nature uses almost exclusively the *L* form of the nineteen common chiral amino acids. The title compound is achiral due to the symmetry of the alpha carbon atom. It also lacks a hydrogen atom on the alpha carbon atom, which is critical in the racemization of the proteinogenic amino acids (Yamada *et al.*, 1983). Little is known about the mechanism of racemization of amino acids lacking an alpha hydrogen atom (Pizzarello & Groy, 2011). Mechanistic investigation of racemization pathways of appropriate derivatives of the title compound may shed light on the racemization of this class of amino acids. Over eighty amino acids that have been identified in meteorites (Burton *et al.*, 2012; Pizzarello *et al.*, 2006). The title compound has not been detected to date in extraterrestrial sources but a higher analog, cycloleucine, has been reported (Pizzarello *et al.*, 2004). The title compound has been incorporated into peptides for conformational studies on model proteins with 1-aminocyclobutane-1-carboxylic acid residues (Balaji *et al.*, 1995). In addition the title compound has been shown to inhibit the enzyme 1-aminocyclopropane-1-carboxylate synthase, part of the pathway for ethylene production that leads to the ripening and spoilage of fruit (Nakatsuka *et al.*, 1998; Bulantseva *et al.*, 2003). The structures of some related non-proteinogenic amino acids have recently been reported (Butcher *et al.*, 2013; Brewer, *et al.*, 2013).

The structure of the title compound has not been reported to the CCDC but there is a report of its hydrochloride salt as a monohydrate (Chacko & Zand, 1975). In the structure of the title compound the amino acid is in the usual zwitterionic form involving the α carboxylate group and all the the bond lengths and angles are in the normal range for such compounds (Orpen, 1993). The metrical parameters of the title compound and its hydrochloride salt do not differ significantly apart from the C—O bond lengths which are equivalent in the title compound but differ significantly in the hydrochloride salt. The cyclobutane backbone of the amino-acid is disordered over two conformations with occupancies of 0.882 (7) and 0.118 (7). There is extensive N—H···O and O—H···O hydrogen bonding (Table 1) linking the zwitterions into a two-dimensional layered structure lying parallel to (100) (Fig. 2).

### 2. Experimental

1-Aminocyclobutane carboxylic acid was purchased from Sigma Aldrich. Crystals of the title compound were grown by evaporation from an aqueous solution of the achiral amino acid.

### 3. Refinement

H atoms were placed in geometrically idealized positions and constrained to ride on their parent atoms with a C—H distances of 0.98 and 0.99 Å. The protons on the N and O were refined isotropically with the O—H distances for the water H's constrained to be 0.82 Å and the H—O—H angle close to 104.5°. The cyclobutane backbone of the amino-acid was disordered over two conformations with occupancies of 0.882 (7) and 0.118 (7).

### Crystal data

**Table:**

C_5_H_9_NO_2_·H_2_O	*F*(000) = 288
*M**_r_* = 133.15	*D*_x_ = 1.312 Mg m^−^^3^
Monoclinic, *P*2_1_/*c*	Cu *K*α radiation, λ = 1.54184 Å
Hall symbol: -P 2ybc	Cell parameters from 4146 reflections
*a* = 10.25082 (19) Å	θ = 4.1–77.2°
*b* = 6.13117 (9) Å	µ = 0.92 mm^−^^1^
*c* = 10.9209 (2) Å	*T* = 123 K
β = 100.8735 (18)°	Prism, colorless
*V* = 674.05 (2) Å^3^	0.41 × 0.34 × 0.16 mm
*Z* = 4	

### Data collection

**Table:**

Agilent Xcalibur (Ruby, Gemini) diffractometer	1400 independent reflections
Radiation source: Enhance (Cu) X-ray source	1360 reflections with *I* > 2σ(*I*)
Graphite monochromator	*R*_int_ = 0.022
Detector resolution: 10.5081 pixels mm^-1^	θ_max_ = 77.4°, θ_min_ = 8.3°
ω scans	*h* = −12→12
Absorption correction: multi-scan (*CrysAlis PRO*; Agilent, 2012)	*k* = −6→7
*T*_min_ = 0.784, *T*_max_ = 1.000	*l* = −13→13
4405 measured reflections	

### Refinement

**Table:**

Refinement on *F*^2^	Secondary atom site location: difference Fourier map
Least-squares matrix: full	Hydrogen site location: inferred from neighbouring sites
*R*[*F*^2^ > 2σ(*F*^2^)] = 0.038	H atoms treated by a mixture of independent and constrained refinement
*wR*(*F*^2^) = 0.103	*w* = 1/[σ^2^(*F*_o_^2^) + (0.0616*P*)^2^ + 0.2169*P*] where *P* = (*F*_o_^2^ + 2*F*_c_^2^)/3
*S* = 1.06	(Δ/σ)_max_ < 0.001
1400 reflections	Δρ_max_ = 0.36 e Å^−^^3^
119 parameters	Δρ_min_ = −0.19 e Å^−^^3^
0 restraints	Extinction correction: *SHELXL97* (Sheldrick, 2008), Fc^*^=kFc[1+0.001xFc^2^λ^3^/sin(2θ)]^-1/4^
Primary atom site location: structure-invariant direct methods	Extinction coefficient: 0.016 (4)

### Special details

**Table:**

Experimental. Absorption correction: CrysAlisPro (Agilent, 2012) Empirical absorption correction using spherical harmonics, implemented in SCALE3 ABSPACK scaling algorithm.
Geometry. All e.s.d.'s (except the e.s.d. in the dihedral angle between two l.s. planes) are estimated using the full covariance matrix. The cell e.s.d.'s are taken into account individually in the estimation of e.s.d.'s in distances, angles and torsion angles; correlations between e.s.d.'s in cell parameters are only used when they are defined by crystal symmetry. An approximate (isotropic) treatment of cell e.s.d.'s is used for estimating e.s.d.'s involving l.s. planes.
Refinement. Refinement of *F*^2^ against ALL reflections. The weighted *R*-factor *wR* and goodness of fit *S* are based on *F*^2^, conventional *R*-factors *R* are based on *F*, with *F* set to zero for negative *F*^2^. The threshold expression of *F*^2^ > σ(*F*^2^) is used only for calculating *R*-factors(gt) *etc*. and is not relevant to the choice of reflections for refinement. *R*-factors based on *F*^2^ are statistically about twice as large as those based on *F*, and *R*- factors based on ALL data will be even larger.

### Fractional atomic coordinates and isotropic or equivalent isotropic displacement parameters (Å^2^)

**Table:**

	*x*	*y*	*z*	*U*_iso_*/*U*_eq_	Occ. (<1)
O1W	0.11907 (10)	0.30045 (14)	0.47599 (9)	0.0268 (3)	
H1W1	0.1576 (18)	0.203 (3)	0.5293 (18)	0.037 (4)*	
H1W2	0.0713 (19)	0.231 (3)	0.4210 (19)	0.039 (5)*	
O2	0.04488 (8)	0.54177 (13)	0.20493 (7)	0.0218 (3)	
O1	0.23745 (8)	0.52875 (13)	0.13611 (7)	0.0213 (3)	
N1	0.13993 (9)	0.73526 (15)	0.41449 (8)	0.0159 (3)	
H1N	0.0661 (18)	0.809 (3)	0.3735 (16)	0.032 (4)*	
H2N	0.1799 (15)	0.815 (3)	0.4840 (15)	0.023 (4)*	
H3N	0.1179 (16)	0.603 (3)	0.4404 (15)	0.026 (4)*	
C1	0.16664 (10)	0.57917 (16)	0.21410 (9)	0.0155 (3)	
C2	0.23577 (10)	0.70405 (17)	0.32992 (9)	0.0151 (3)	
C3	0.30893 (12)	0.9136 (2)	0.30010 (12)	0.0247 (3)	
H3A	0.2845 (16)	0.967 (3)	0.2165 (16)	0.030*	
H3B	0.3019 (16)	1.021 (3)	0.3606 (16)	0.030*	
C4A	0.44072 (14)	0.7839 (3)	0.32757 (17)	0.0353 (6)	0.882 (7)
H4AA	0.4706	0.7309	0.2518	0.042*	0.882 (7)
H4AB	0.5134	0.8607	0.3837	0.042*	0.882 (7)
C4B	0.4308 (11)	0.845 (2)	0.3984 (14)	0.0353 (6)	0.118
H4BA	0.4366	0.9176	0.4802	0.042*	0.118 (7)
H4BB	0.5162	0.8543	0.3689	0.042*	0.118 (7)
C5	0.37127 (10)	0.6082 (2)	0.39333 (11)	0.0228 (3)	
H5A	0.3873 (16)	0.464 (3)	0.3693 (15)	0.027*	
H5B	0.3840 (16)	0.615 (3)	0.4822 (16)	0.027*	

### Atomic displacement parameters (Å^2^)

**Table:**

	*U*^11^	*U*^22^	*U*^33^	*U*^12^	*U*^13^	*U*^23^
O1W	0.0356 (5)	0.0181 (4)	0.0236 (5)	−0.0006 (3)	−0.0028 (4)	0.0030 (3)
O2	0.0172 (4)	0.0236 (5)	0.0229 (4)	−0.0017 (3)	−0.0010 (3)	−0.0053 (3)
O1	0.0237 (4)	0.0252 (5)	0.0150 (4)	0.0027 (3)	0.0035 (3)	−0.0017 (3)
N1	0.0166 (5)	0.0170 (5)	0.0139 (5)	0.0002 (3)	0.0023 (3)	−0.0002 (3)
C1	0.0186 (5)	0.0131 (5)	0.0134 (5)	0.0021 (4)	−0.0006 (4)	0.0021 (4)
C2	0.0143 (5)	0.0163 (5)	0.0141 (5)	0.0002 (4)	0.0017 (4)	0.0004 (4)
C3	0.0271 (6)	0.0214 (6)	0.0273 (6)	−0.0085 (4)	0.0098 (5)	−0.0027 (5)
C4A	0.0195 (8)	0.0404 (10)	0.0476 (11)	−0.0086 (6)	0.0105 (7)	−0.0069 (7)
C4B	0.0195 (8)	0.0404 (10)	0.0476 (11)	−0.0086 (6)	0.0105 (7)	−0.0069 (7)
C5	0.0148 (5)	0.0313 (7)	0.0204 (6)	0.0039 (4)	−0.0018 (4)	−0.0033 (4)

### Geometric parameters (Å, º)

**Table:**

O1W—H1W1	0.87 (2)	C3—C4A	1.547 (2)
O1W—H1W2	0.82 (2)	C3—H3A	0.958 (17)
O2—C1	1.2543 (13)	C3—H3B	0.943 (18)
O1—C1	1.2574 (13)	C4A—C5	1.542 (2)
N1—C2	1.4823 (13)	C4A—H4AA	0.9900
N1—H1N	0.922 (18)	C4A—H4AB	0.9900
N1—H2N	0.930 (17)	C4B—C5	1.570 (12)
N1—H3N	0.902 (17)	C4B—H4BA	0.9900
C1—C2	1.5330 (14)	C4B—H4BB	0.9900
C2—C5	1.5465 (14)	C5—H5A	0.942 (17)
C2—C3	1.5527 (15)	C5—H5B	0.956 (17)
C3—C4B	1.545 (13)		
			
H1W1—O1W—H1W2	105.5 (18)	C2—C3—H3B	108.9 (10)
C2—N1—H1N	109.9 (11)	H3A—C3—H3B	112.9 (14)
C2—N1—H2N	109.5 (9)	C5—C4A—C3	89.22 (9)
H1N—N1—H2N	109.6 (14)	C5—C4A—H4AA	113.8
C2—N1—H3N	108.3 (10)	C3—C4A—H4AA	113.8
H1N—N1—H3N	111.2 (15)	C5—C4A—H4AB	113.8
H2N—N1—H3N	108.2 (14)	C3—C4A—H4AB	113.8
O2—C1—O1	126.43 (10)	H4AA—C4A—H4AB	111.0
O2—C1—C2	117.09 (9)	C3—C4B—C5	88.3 (6)
O1—C1—C2	116.46 (9)	C3—C4B—H4BA	113.9
N1—C2—C1	108.72 (8)	C5—C4B—H4BA	113.9
N1—C2—C5	114.52 (8)	C3—C4B—H4BB	113.9
C1—C2—C5	114.58 (9)	C5—C4B—H4BB	113.9
N1—C2—C3	115.28 (9)	H4BA—C4B—H4BB	111.1
C1—C2—C3	113.99 (9)	C4A—C5—C2	88.88 (9)
C5—C2—C3	88.84 (8)	C2—C5—C4B	88.6 (4)
C4B—C3—C2	89.3 (4)	C4A—C5—H5A	113.8 (10)
C4A—C3—C2	88.44 (9)	C2—C5—H5A	114.8 (10)
C4B—C3—H3A	142.0 (11)	C4B—C5—H5A	141.8 (11)
C4A—C3—H3A	115.0 (10)	C4A—C5—H5B	117.2 (9)
C2—C3—H3A	115.6 (10)	C2—C5—H5B	112.2 (10)
C4B—C3—H3B	82.1 (12)	C4B—C5—H5B	86.9 (11)
C4A—C3—H3B	113.6 (10)	H5A—C5—H5B	109.0 (14)
			
O2—C1—C2—N1	5.49 (13)	C2—C3—C4A—C5	−16.15 (9)
O1—C1—C2—N1	−176.10 (9)	C4A—C3—C4B—C5	−71.5 (8)
O2—C1—C2—C5	135.05 (10)	C2—C3—C4B—C5	16.8 (5)
O1—C1—C2—C5	−46.54 (13)	C3—C4A—C5—C2	16.21 (10)
O2—C1—C2—C3	−124.62 (10)	C3—C4A—C5—C4B	−72.9 (8)
O1—C1—C2—C3	53.79 (13)	N1—C2—C5—C4A	−133.55 (10)
N1—C2—C3—C4B	99.7 (6)	C1—C2—C5—C4A	99.82 (11)
C1—C2—C3—C4B	−133.6 (6)	C3—C2—C5—C4A	−16.16 (10)
C5—C2—C3—C4B	−17.0 (6)	N1—C2—C5—C4B	−100.6 (6)
N1—C2—C3—C4A	132.80 (10)	C1—C2—C5—C4B	132.7 (6)
C1—C2—C3—C4A	−100.42 (11)	C3—C2—C5—C4B	16.8 (6)
C5—C2—C3—C4A	16.10 (10)	C3—C4B—C5—C4A	73.3 (8)
C4B—C3—C4A—C5	74.9 (8)	C3—C4B—C5—C2	−16.9 (5)

### Hydrogen-bond geometry (Å, º)

**Table:**

*D*—H···*A*	*D*—H	H···*A*	*D*···*A*	*D*—H···*A*
O1*W*—H1*W*1···O1^i^	0.87 (2)	1.92 (2)	2.7935 (12)	175.2 (18)
O1*W*—H1*W*2···O2^ii^	0.82 (2)	2.01 (2)	2.8268 (12)	175.7 (19)
N1—H1*N*···O2^iii^	0.922 (18)	1.923 (18)	2.8087 (12)	160.6 (15)
N1—H2*N*···O1^iv^	0.930 (17)	1.913 (17)	2.8351 (12)	171.2 (14)
N1—H3*N*···O1*W*	0.902 (17)	1.895 (17)	2.7673 (13)	162.3 (15)

Symmetry codes: (i) *x*, −*y*+1/2, *z*+1/2; (ii) −*x*, *y*−1/2, −*z*+1/2; (iii) −*x*, *y*+1/2, −*z*+1/2; (iv) *x*, −*y*+3/2, *z*+1/2.
